# Assessment of the dose-dependent biochemical and cytotoxicity of zein-coated MgO nanowires in male and female albino rats

**DOI:** 10.1080/07853890.2021.1991587

**Published:** 2021-10-25

**Authors:** Ghada H. Naguib, Gamal S. Abd El-Aziz, Hisham A. Mously, Sahar M. Bukhary, Mohamed T. Hamed

**Affiliations:** a Department of Restorative Dentistry, Faculty of Dentistry, King Abdulaziz University, Jeddah, Saudi Arabia; b Department of Oral Biology, Faculty of Dentistry, Cairo University, Cairo, Egypt; c Department of Anatomy, Faculty of Medicine, King Abdulaziz University, Jeddah, Saudi Arabia; d Department of Oral and Maxillofacial Prosthodontics, Faculty of Dentistry, King Abdulaziz University, Jeddah, Saudi Arabia; e Department of Oral Biology, Faculty of Dentistry, King Abdulaziz University, Jeddah, Saudi Arabia; f Department of Fixed Prosthodontics, Faculty of Dentistry, Cairo University, Cairo, Egypt

**Keywords:** Zein, MgO nanowires, liver, kidney, rats

## Abstract

**Introduction:** Recently, zein-coated MgO nanowires were synthesized, which could be promising as an effective antimicrobial compounds that can be combined in the preparation of a diversity of new dental formulations. However, there is a deficiency of information concerning their toxicological profile regarding the human health.

**Objective:** This in vivo study aimed to explore the hepato- and nephrotoxicity of low versus high doses of zein-coated MgO nanowires in rats.

**Materials and Methods:** A 21-day recurrent dose toxicity research was carried out. Wistar rats were divided into 2 main groups, males and females (*n* = 18). Each group was further subdivided into 3 subgroups: control, MgO-zein nanowires low dose, MgO-zein nanowires high dose. The low dose used was 100 mg/kg while the high dose used was 200 mg/kg.

**Results:** The results showed that MgO-zein nanowires at both doses did not affect the electrolytes levels compared to the control levels. Also, they did not produce any significant alteration in liver function markers in both rats' genders. MgO-zein nanowires at both doses did not produce any effective alteration in serum creatinine in treated rats of both genders. Moreover, very minimal histological alterations were observed in both doses of MgO-zein nanowires in liver and kidney of both genders.

**Conclusion:** Based on the observed safety of zein-coated MgO nanowires, it can be utilized as an effective antimicrobial compound that can be combined in the preparation of a diversity of new dental formulations.

**KEY MESSAGES**
- MgO NPs are globally used in multiple fields including the therapeutic field.
- Zein has wide pharmaceutical applications especially coating the tablet over sugar.
- There are no cytotoxic studies that investigate MgO-zein nanowires safety until now.

## Introduction

Nanoparticles (NPs) are substances that have a single-dimensional character in the range of 1–100 nm that make them suitable for many applications including industry, agriculture, medicine, and therapeutics [1–3]. Different new mechanical, electrical, optical, thermal, and magnetic properties are manifested with the small size of NPs [4–6].

Amongst the prominent metal oxides NPs, magnesium oxide (MgO) NPs have drawn broad scientific concerns because it is simply synthesized and chemically stable compound. MgO NPs are globally used in multiple fields including the therapeutic field [7] where they are utilized as an antacid, detoxifying preparation, and biomolecular diagnostics in addition to their significant bactericidal action and tumour inhibition [8–10]. However, with their increased application in daily life, their toxic effect on the environment and human health is of concern [11]. Some *in vitro* and *in vivo* cytotoxicity studies performed on MgO NPs have documented its toxic effect where an investigation demonstrated that treatment of human cardiac endothelial cells with MgO NPs, resulted in a time and concentration-dependent cytotoxicity [12]. In another study, intratracheal instillation of MgO NPs to the rat lungs resulted in a dose-dependent increase in lung tissue destruction markers and histopathology [13]. Also, it was reported that oral administration of high concentrations of MgO NPs in female albino rats resulted in significant DNA destruction and increased levels of kidney and liver markers enzymes. The authors attributed these biochemical alterations to Mg accumulation in the liver and kidney tissues [14].

The one-dimensional shape of nanowires opens the door to different applications along with electronics, biomedicine, and optoelectronics [15–18]. They can also increase the use of variable materials when applied as a template to grow the core of their heterostructures [19].

It was reported that the thermal stability, chemical inertness, and excellent electrical insulating characteristics of MgO make it appealing to be used as a nanowire template [20]. Unfortunately, MgO nanowires were found relatively insoluble and tend to agglomerate, deteriorating the antibacterial properties of the nanowires [7]. This made us consider a surfactant-based stabilizer for MgO nanowires that not just prevents the agglomeration but even facilitates their dissolution to obtain optimum properties.

Zein is a natural alcohol-soluble protein present in the endosperm tissue of corn. It is an amorphous polymer that has a glass transition temperature of 165 °C; giving rise to plasticizing viscoelasticity. This innate property helps zein to stabilize particles against aggregation by greatly reducing the hydrophobic attraction and increasing steric repulsion at the molecular level that can be of great advantage [8,21].

Film coating of medicines and metal nanoparticles, such as Mg oxide (MgO), zein polymer showed encouraging results [22]. It was reported that MgO nanowires have strong bactericidal activity against pathogens and can be added to multiple dental cements to provide antimicrobial activity [23,24]. Recently, a research study conducted by our group showed significant antibacterial characters of 1 and 2% zein-coated MgO nanowires against four organisms, *Staphylococcus aureus*, *Streptococcus mutans*, *Enterococcus faecalis*, and *Candida albicans*. Therefore, they could be incorporated into the formulation of a variety of new dental materials and products to improve dental care and oral health [25].

Zein (a corn polymer) is reported for desirable properties, such as biocompatibility, low toxicity, and high absorbability of the degraded end products *in-vivo* [26]. Zein has wide pharmaceutical applications especially coating the tablet over sugar due to its high resistance to heat, humidity, abrasion, and capacity to mask the strong odour or unacceptable taste. Due to water insolubility and swelling properties, zein was even explored for its use in control or sustain the drug release from the dosage form [26,27]. Its unique properties, such as biocompatibility and biodegradability make the polymer a promising candidate in the formation of micro or nanospheres and even film coatings to modify the gene delivery.

However, there are no cytotoxic studies that investigate MgO-zein nanowires safety until now. Therefore, this research aimed to examine the hepato- and nephrotoxicity of low *vs.* high doses of zein-coated MgO nanowires in rats. Furthermore, this study investigated the gender difference in toxicity through a comparison between male and female rats’ results.

## Materials and methods

### Chemicals

Zein polymer and all other chemicals, stains, and kits applied in this study were acquired from the local agent of Sigma–Aldrich in Jeddah, Saudi Arabia.

### Synthesis of MgO nanowires

MgO nanowires were prepared by the direct reaction of magnesium acetate (Mg [CH3COO]_2_) and urea (CH_4_N_2_O) by adopting a microwavable hydrothermal technique [7]. Magnesium acetate was blended with water (6.4 g/75 mL) for 30 min at a controlled condition of 25 °C. Few drops of urea were added into water under continuous stirring conditions (1.2 g/25 mL). Then the mix (100 mL) was sealed in a non-stick autoclave and retained at 180 °C for 15 min. The autoclave was supported with a microwave furnace (1000 W). Upon cooling of the autoclave the products were collected and filtered using distilled water, rinsed with ethanol, and finally dehydrated at 60 °C for 24 h. The obtained product was then calcinated at 600 °C for 1 h.

### Zein coating of MgO-nanowires

Coating of MgO nanowires with zein was developed as described in our previous work [22]. Briefly, a mix of 0.02 g of zein ethanol and 0.1 NaOH solutions (93.7% (v/v) was prepared. With ultrasonic shear (750 W and 20 kHz frequency) drops of zein solution were added into a 15 ml solution of 0.02 g Of MgO and polyvinyl alcohol [0.9% (w/v)]. An ice bath was used to keep the temperature at 10 °C. Afterwards, the mix was stirred at 500 rpm at 37 °C till ethanol evaporation. Then excess PVA and the suspension of nanowires with zein were centrifuged twice at 3000 rpm for 45 min and the pellet was re-dispersed in 5 ml buffer [28]. Zein polymer was then mixed with polyvinyl alcohol (PVA) at 2:1 proportion by weight and MgO nanowires at a proportion by weight of 4:1. MgO-zein and PVa solution was mixed on a magnetic stirrer for a period of 30 min. After separation of the MgO-zein nanowires, the mix was left to evaporate the PVA, then centrifuged and freeze-dried. Before the rat injection, MgO-zein nanowires were suspended in Milli-Q water and then ultrasonicated and vortexed.

### Characterization of MgO-zein nanowires

Transmission Electron Microscope (TEM) and Scanning Electron microscope (SEM) examination in our previous study revealed that the MgO-zein nanowires were 100 nm thick and 1–2 µm long compared to pure uncoated MgO nanowires of 50–60 nm wide and 500–1000 nm long [22]. Zein coating was well-adsorbed over the MgO nanowires due to the electrostatic attraction between the opposite charges of zein and MgO [29]. Similarly, Differential scanning calorimetry (DSC) and Fourier Transform infra-red spectroscopy (FTIR) indicated the formation of a stable coating with no new component formation [22,30].

### Animals

All animals were acquired from King Fahd Medical Research Centre (KFMRC), King Abdulaziz University, Saudi Arabia. Experiments were approved by King Abdulaziz University, Saudi Arabia Animal Research Ethics Board (Ref 716-19).

This study was conducted on 36 (18 males and 18 females) Wistar albino rats (weighing 160–180 g). Animals were fed classic lab food and water and were caged at a humidity of 50–60%, a temperature of 22 °C, and taken into consideration the 12 h light/12 h dark cycle. Handling of animals was in strict accordance with the ARRIVE guidelines.

### Research design

Rats were casually split into two main groups, males (*n* = 18) and females (*n* = 18). Both groups were further subdivided into three subgroups; (1) Control males, (2) MgO-zein nanowires low dose treated males, (3) MgO-zein nanowires high dose treated males, (4) Control females, (5) MgO-zein nanowires low dose treated females, and (6) MgO-zein nanowires high dose treated females. The male and female control groups were injected intraperitoneally (i.p.) with Milli-Q water/day while the male and female MgO-zein nanowires low dose treated groups were injected i.p. with 100 mg/kg body weight/day and the male and female MgO-zein nanowires high dose treated groups were injected i.p. with 200 mg/kg body weight/day weight. Doses were chosen based on our previous pilot study.

### Blood and tissue collection

After 21 days from the beginning of the experiment and overnight fasting, the rats were sacrificed by an overdose of ether. Immediately blood samples were taken from the retrorbital venous plexus using the heparinized tube, sera were separated by centrifugation at 3000 rpm for 10 min, then frozen and saved at −70 °C for biochemical studies. Afterwards, the abdominal cavity of each rat was opened where the liver and both kidneys were removed and cut into small pieces and immediately preserved for histopathological study in 10% neutral buffer formalin (NBF).

### Biochemical investigation

The serum electrolytes (sodium, potassium, chloride, and iodide), enzymes of liver functions: alanine aminotransferase (ALT), aspartate aminotransferase (AST), alkaline phosphatase (ALP), and total bilirubin (TB), kidney activity markers, such as blood urea nitrogen (BUN) plus creatinine were quantified using SIEMENS Dimension Vista System, King Abdulaziz Hospital, KAU, Jeddah, Saudi Arabia.

### Histopathological study

The formalin-fixed liver and kidney pieces were processed through graded alcohols and xylene and embedded in paraffin blocks in the automatic processor of the histopathology lab. Serial 5 µm slices were prepared and stained with haematoxylin and eosin (H & E) to assess the general structure [31]. The slides were blindly examined under a light microscope by an expert pathologist. Photography of the slides from different groups at variable magnifications was done using a camera-loaded microscope Olympus BX53 (Olympus, Tokyo, Japan).

### Analysis of data

Data was conferred by the mean ± standard deviation (SD). Comparison among groups was carried out using ANOVA and *post-hoc* Tukey HSD tests to determine the significant differences among different groups utilizing SPSS version 22.

## Results

### Effect of MgO-zein nanowires injection on serum electrolytes quantified in male and female rats

**Figure 1.**
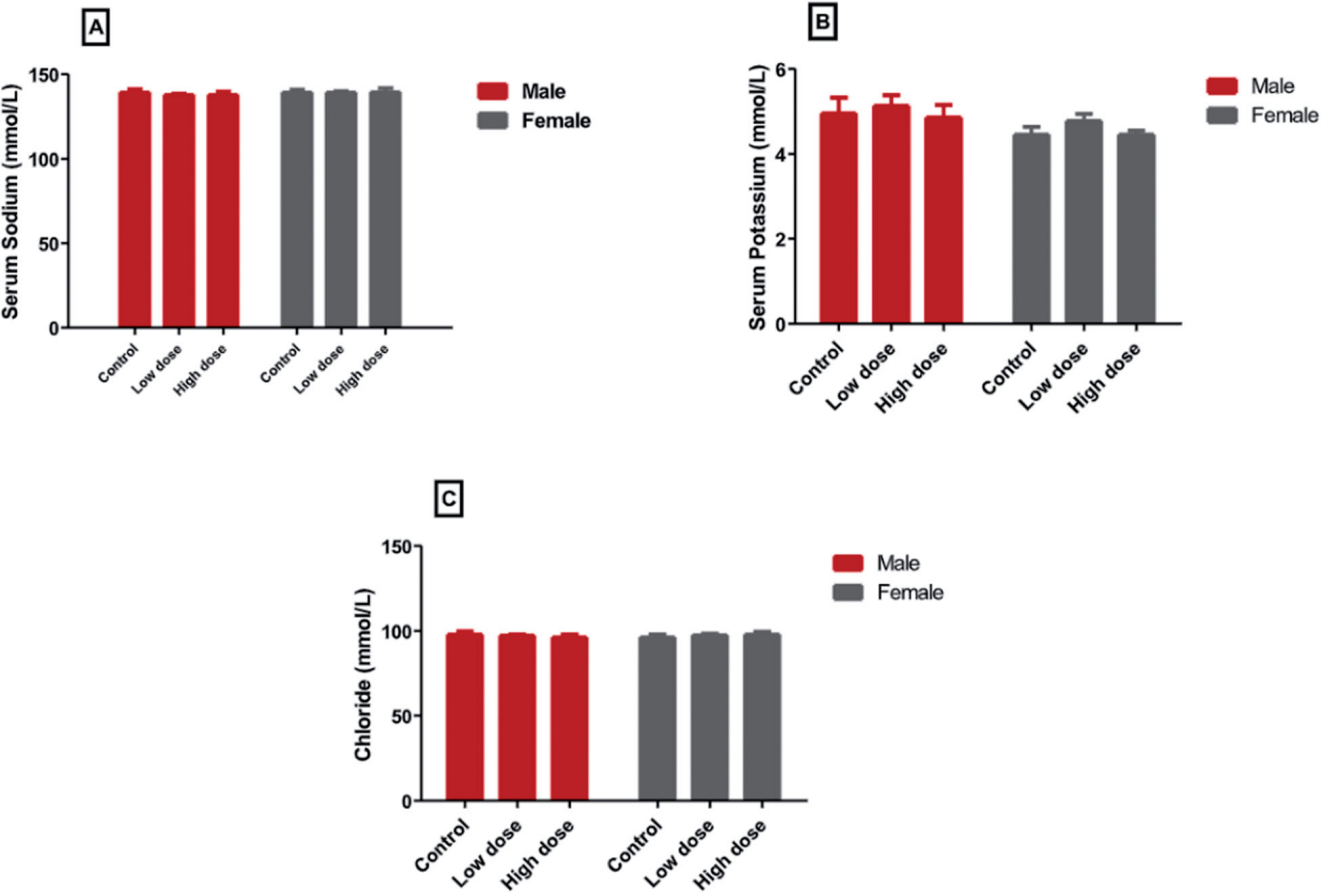
Effect of low and high doses (100 and 200 mg/kg, respectively). MgO-zein nanowires on serum levels of different electrolytes measured in male and female rats. Results are shown as mean ± *SD* (*n* = 5). ^#^Significance at *p* ≤ .05 using One-way ANOVA succeeded by Tukey HSD test.

Both low (100 mg/kg) and high (200 mg/kg) doses of MgO-zein nanowires did not show significant changes in the mean values of sodium, potassium, and chloride ions *vs.* the control values, both in male and female rats (Figure 1(A–C)). Regarding the sodium (Figure 1(A)), the low dose resulted in more or less similar values (137.8 ± 1.8 and 139.2 ± 1.4 [mmol/L] in male and female rats, respectively) as compared to control (139.2 ± 1.92 and 139.2 ± 1.64 [mmol/L] in males and females, respectively). Also, the high dose did not show significant changes (137.8 ± 1.75 and 139.4 ± 2. 34 [mmol/L] in male and female rats, respectively) compared to control values. Regarding the potassium (Figure 1(B)), the low dose resulted in a very slight increase (5.1 ± 0.3 and 4.7 ± 0.2 [mmol/L] in male and female rats, respectively) as compared to the control values (4.96 ± 0.37 and 4.46 ± 0.18 [mmol/L] in male and female rats, respectively) while in high dose (4.86 ± 0.29 and 4.46 ± 0.09 [mmol/L] in male and female rats, respectively), there was nearly no changes from the control values.

In the case of chloride ions (Figure 1(C)), both low dose (97.4 ± 0.6 and 97.6 ± 1.8 [mmol/L] in male and female rats, respectively) and high dose (96.46 ± 1.52 and 98.14 ± 1.73 [mmol/L] in male and female rats, respectively) did not cause significant changes from the control group (98.06 ± 1.87 in males and 96.44 ± 1.52 [mmol/L] females).

### Effect of MgO-zein nanowires injection on serum liver function markers quantified in male and female rats

**Figure 2.**
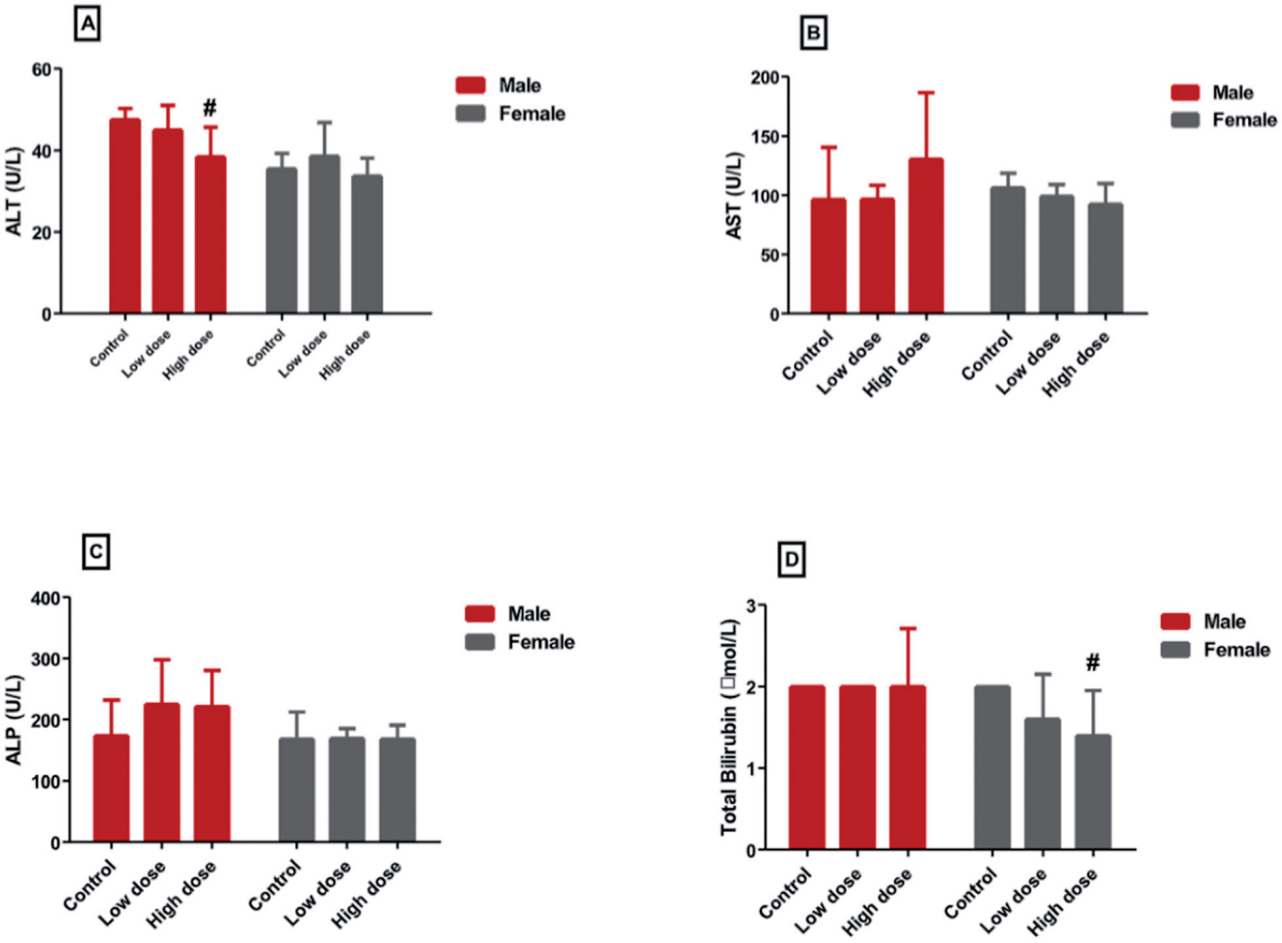
Effect of low and high doses (100 and 200 mg/kg, respectively). MgO-zein nanowires on serum levels of liver function markers measured in male and female rats. Results are shown as mean ± *SD* (*n* = 5). ^#^Significance at *p* ≤ .05 using One-way ANOVA succeeded by Tukey HSD test.

Figure 2 displayed the effect of low and high doses MgO-zein nanowires on liver enzymes and bilirubin in male and female rats. It could be seen that low dose produced no alteration in ALT (45.02 ± 5.9 and 38.6 ± 8.1 [U/L] in male and female rats, respectively), AST (96.8 ± 11.4 and 99.4 ± 9.7 [U/L] in male and female rats, respectively), ALP (225.6 ± 32.4 and 169.6 ± 16.3 [U/L] in male and female rats, respectively), and bilirubin (2.6 ± 0.6 and1.6 ± 0.4 [U/L] in male and female rats, respectively) as compared to their respective values in control rats (47.22 ± 2.7 and 35.6 ± 3.7 [U/L] in male and female rats), (96.4 ± 16.9 and 106.4 ± 11.1 [U/L] in male and female rats), (174.4 ± 26.9 and 168.8 ± 23.2 [U/L] in male and female rats), and (2.4 ± 0.9 and 2.8 ± 0.12 [U/L] in male and female rats). However, it was found that the high dose of MgO-zein nanowires significantly decreased serum ALT level (38.4 ± 2.3 and 33.6 ± 4.3 [U/L] in male and female rats, respectively) compared to the respective controls. Furthermore, the high dose significantly decreased serum bilirubin (1.9 ± 0.7 and 1.4 ± 0.3 [U/L] in male and female rats, respectively) in comparison to the respective control.

### Effect of MgO-zein nanowires injection on serum kidney function markers quantified in both rats genders

**Figure 3.**
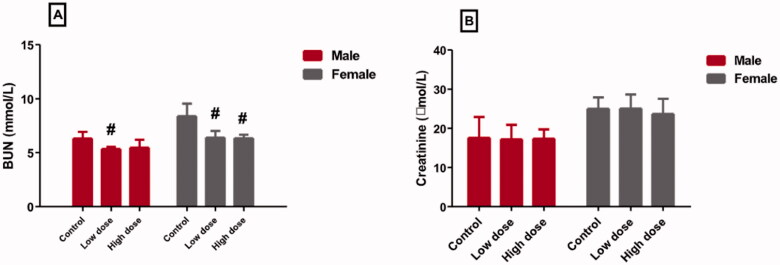
Effect of low and high doses (100 and 200 mg/kg, respectively). MgO-zein nanowires on serum levels of kidney function markers measured in male and female rats. Results are shown as mean ± *SD* (*n* = 5). ^#^Significance at *p* ≤ .05 using One-way ANOVA succeeded by Tukey HSD test.

Injection of a low dose of MgO-zein nanowires in both genders produced a significant decrease in serum BUN level (5.4 ± 0.2 and 6.3 ± 0.6 [mmol/L] in male and female rats, respectively) compared to control (6.3 ± 0.6 and 8.4 ± 1.14 [mmol/L] in male and female rats, respectively). Also, the high dose slightly decreased the serum BUN level (5.8 ± 0.7 and 6.3 ± 0.3 [mmol/L] in male and female rats, respectively) (Figure 3(A)). Moreover, it was found both doses of MgO-zein nanowires (17.4 ± 3.2 and 25.3 ± 3.6 [mmol/L] in male and female rats for low dose), (17.5 ± 2.5 and 23.7 ± 3.8 [mmol/L] in male and female rats for high dose) produced no detectable changes in the serum creatinine levels *vs.* to their respective control rats (17.6 ± 2.6 and 25.4 ± 2.9 [mmol/L] in male and female rats, respectively) (Figure 3(B)).

### Effect of MgO-zein nanowires injection on liver histopathology in both rats gender

**Figure 4.**
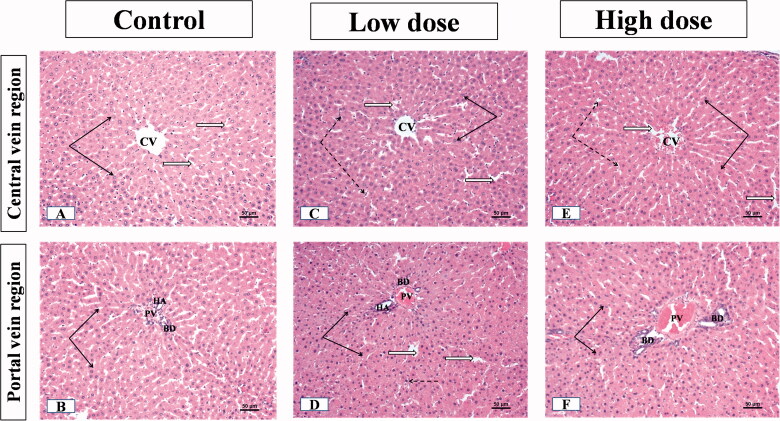
Illustrative photographs displaying the effect of low and high doses (100 and 200 mg/kg, respectively). MgO-zein nanowires on liver histopathology in male rats (H & E stain × 200). Control group (A,B) showed A: normal central vein (CV), hepatocyte cell cords (black arrows), separated by narrow sinusoids (white arrows), cell nuclei are rounded and vesicular; B: normal portal area contents [bile duct (BD), hepatic artery (HA), and branches of portal vein (PV)]. Low dose group (C,D) showed (C) normal hepatic architecture with the normal central vein (CV) and hepatocyte cell cords (arrows), blood sinusoids showed slight dilation (white arrows) with prominent Kupffer cells (dotted arrows); (D) Slight congestion and dilatation of portal vein (PV), the proliferation of bile ducts (BD) and slight proliferation of connective tissue were seen in the portal area (white star) while nearby hepatocytes showed active vesicular nuclei (arrows). High dose group (E,F) showed (E) no alteration of hepatic parenchyma normal slightly dilated central vein (CV), normal hepatocytes with active nuclei (black arrows), prominent Kupffer cells (dotted arrows); (F) portal area showed proliferation of bile duct (BD) and slight congestion of portal veins (PV), besides vessel congestion with an increase in periductal connective tissue (stars) while nearby hepatocytes showed active vesicular nuclei (arrows).

The examination of liver sections from male rats showed that the control group displayed the normal liver parenchyma where hepatocytes were emitted from the central vein towards portal areas in cords. The threads were spaced by endothelial lined narrow blood sinusoids. Hepatocytes nuclei were rounded, vesicular and central in position (occasionally, some cells were binucleated) (Figure 4(A)). The portal regions showed the bile duct, hepatic artery, and portal vein branches (Figure 4(B)). In the group receiving low dose (100 mg/kg) MgO-zein nanowires, the liver parenchyma showed normal architecture. The hepatocytes looked normal with vesicular active nuclei and homogenous cytoplasm while few cells showed nuclear enlargement or karyomegaly with slight dilatation of blood sinusoids (Figure 4(C)). Slight dilatation and congestion of portal vein, the proliferation of bile ducts, and slight proliferation of connective tissue were seen in the portal area (Figure 4(D)).

The liver of male rats receiving high dose (200 mg/kg) MgO-zein nanowires showed also normal hepatic parenchyma. The hepatocytes near the central vein showed a normal appearance while few scattered cells showed dark nuclei (Figure 4(E)). Some portal areas showed slight portal vein congestion, the proliferation of bile duct with an increase in periductal connective tissue (Figure 4(F)).

**Figure 5.**
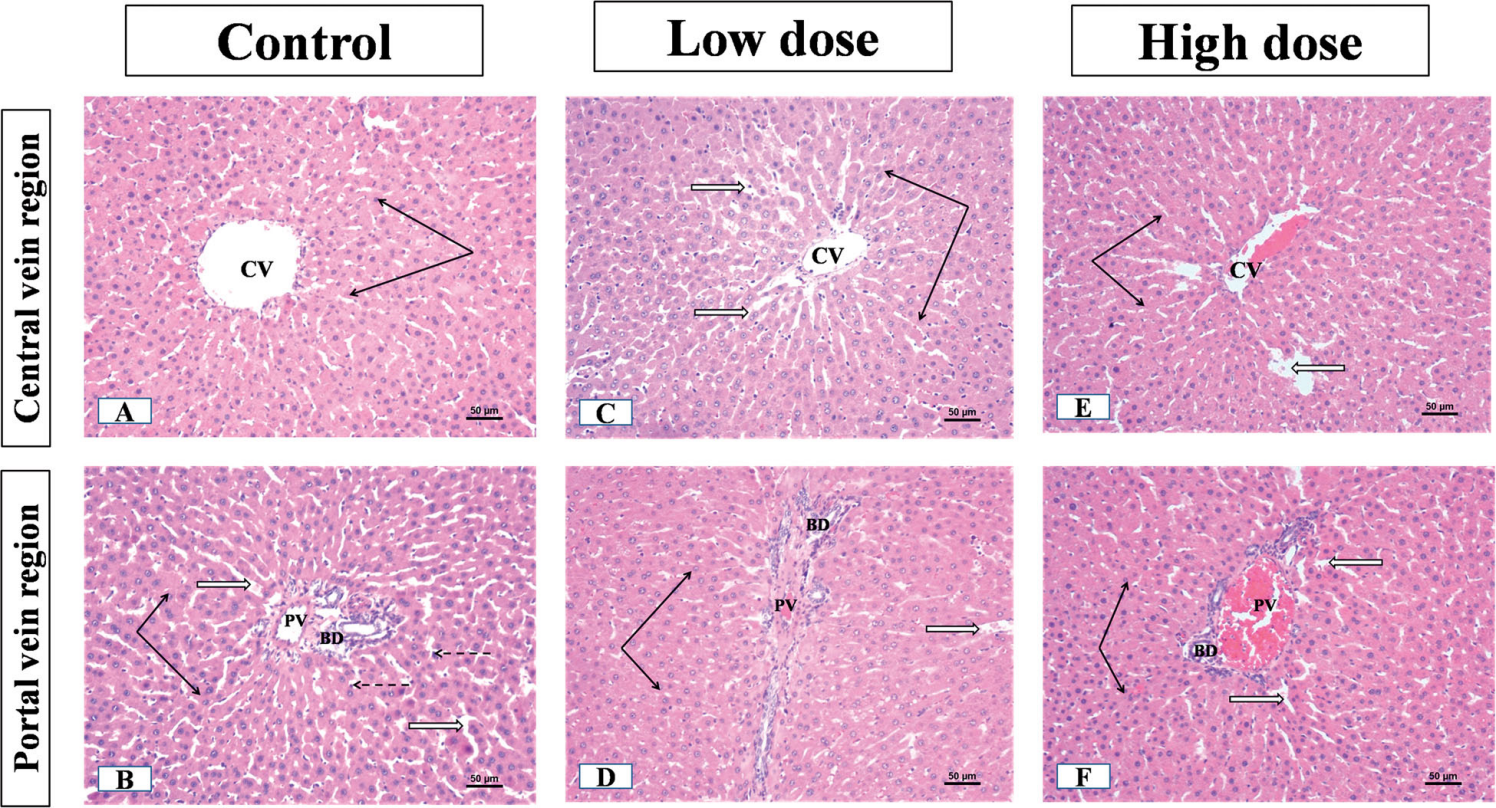
Illustrative photographs displaying the effect of low and high doses (100 and 200 mg/kg, respectively). MgO-zein nanowires on liver histopathology in female rats (H & E stain × 200). Control group (A,B) showed (A) normal central vein (CV), hepatocyte cell cords (black arrows), cell nuclei are rounded and vesicular; (B) normal portal vein (PV). Low dose group (C,D) showed (C) normal hepatocytes cell cords with their rounded active vesicular nuclei (black arrows); (D) portal regions (PV) showed slight congestion of blood portal veins, normal bile ducts, and connective tissue. High dose group (E,F) showed (E) hepatocytes with slightly small nuclei and condensed chromatin at central vein region (black arrows); (F) hepatocytes with slightly small nuclei and condensed chromatin at portal veins (PV) regions (black arrows).

The examination of the liver sections from female rats showed that the control group did not show any significant difference as compared to that described for the male group showing normal cytoplasm and vesicular nuclei divided by thin wall blood sinusoids in hepatocytes (Figure 5(A)). The portal area showed normal contents (artery, vein, and bile ducts) like those described in male rats’ liver (Figure 5(B)). The minimal alteration was seen in the hepatic parenchyma of livers from the low dose (100 mg/kg) MgO-zein nanowires group, as the liver showed normal central and portal blood vessels. The hepatocytes cell cords appeared normal showing spherical active vesicular nuclei. Portal regions showed negligible veins congestion, normal bile ducts, and connective tissue (Figure 5(C,D)). In the high dose (200 mg/kg) MgO-zein nanowires group, the hepatocytes showed slightly small nuclei with condensed chromatin at central as well as portal veins regions (Figure 5(E,F)).

### Effect of MgO-zein nanowires injection on kidney histopathology in male and female rats

**Figure 6.**
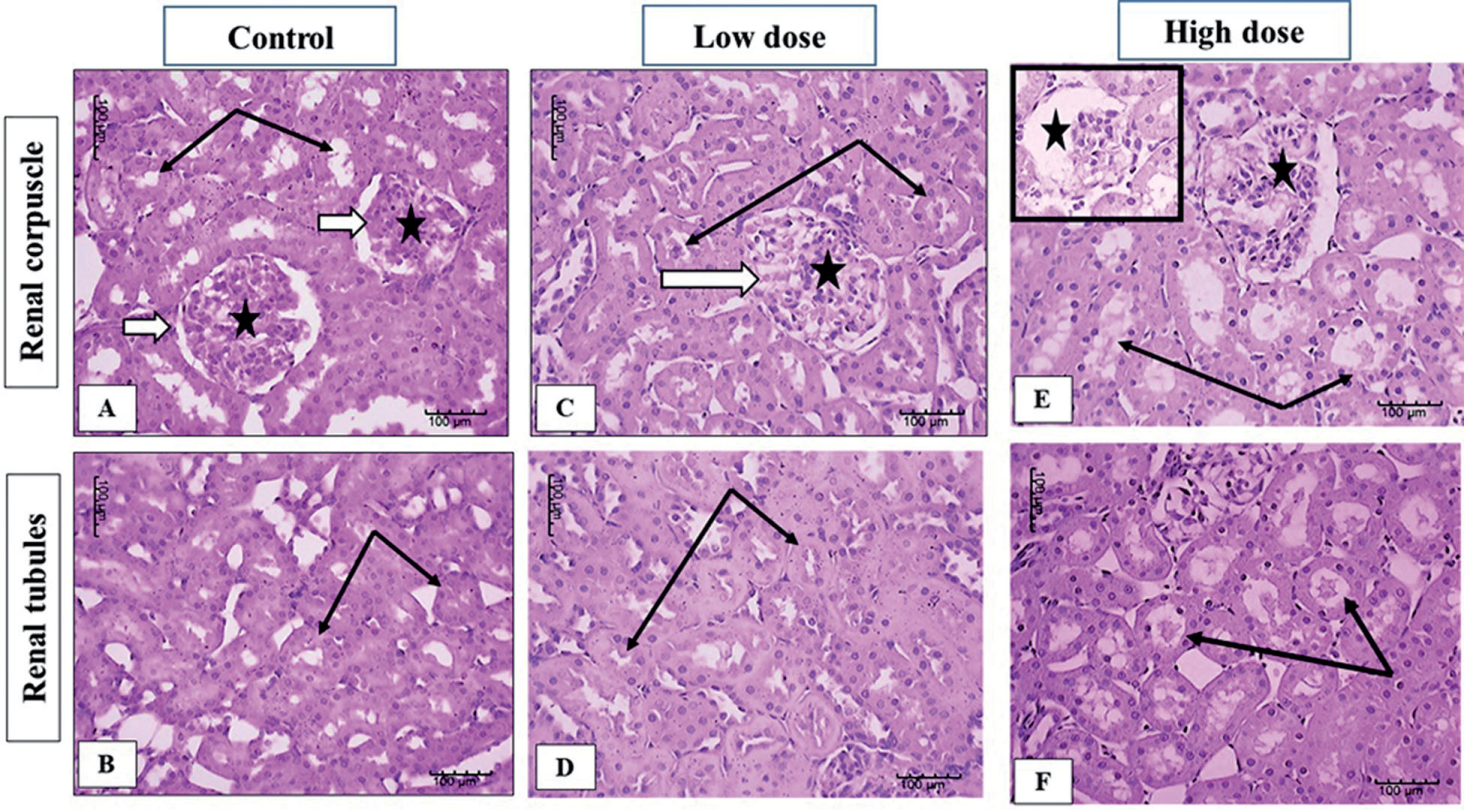
Illustrative photographs displaying the effect of low and high doses (100 and 200 mg/kg, respectively). MgO-zein nanowires on kidney histopathology in male rats (H & E stain × 200). Control group (A,B) showed normal renal corpuscle (white arrow), glomeruli (star), and tubules (black arrows). Low dose group (C,D) showed mild dilation of glomerular capillaries (white arrows and star) and tubules also showed mild dilation. High dose group (E,F) showed focal disorganization or atrophy of glomerular capillaries (stars), and tubules are dilated and contain scanty hyaline material (black arrows).

The examination of the kidney sections from male rats showed that the cortical and medullary kidney parenchyma of control male rats had the normal organization described in literature concerning renal corpuscles with its glomerular capillaries and tubules with intact epithelial lining (Figure 6(A,B)). In the kidney of a rat receiving low dose MgO-zein nanowires (100 mg/kg) there were mild changes including slight dilation and congestion of glomerular capillaries. Medullary tubules showed also mild dilation but intact lining epithelium (Figure 6(C,D)). However, high dose MgO-zein nanowires (200 mg/kg) administration resulted in focal disorganization or atrophy of glomerular capillaries in some corpuscles. In addition, renal tubules were dilated and contain scanty hyaline material (Figure 6(E,F)).

**Figure 7.**
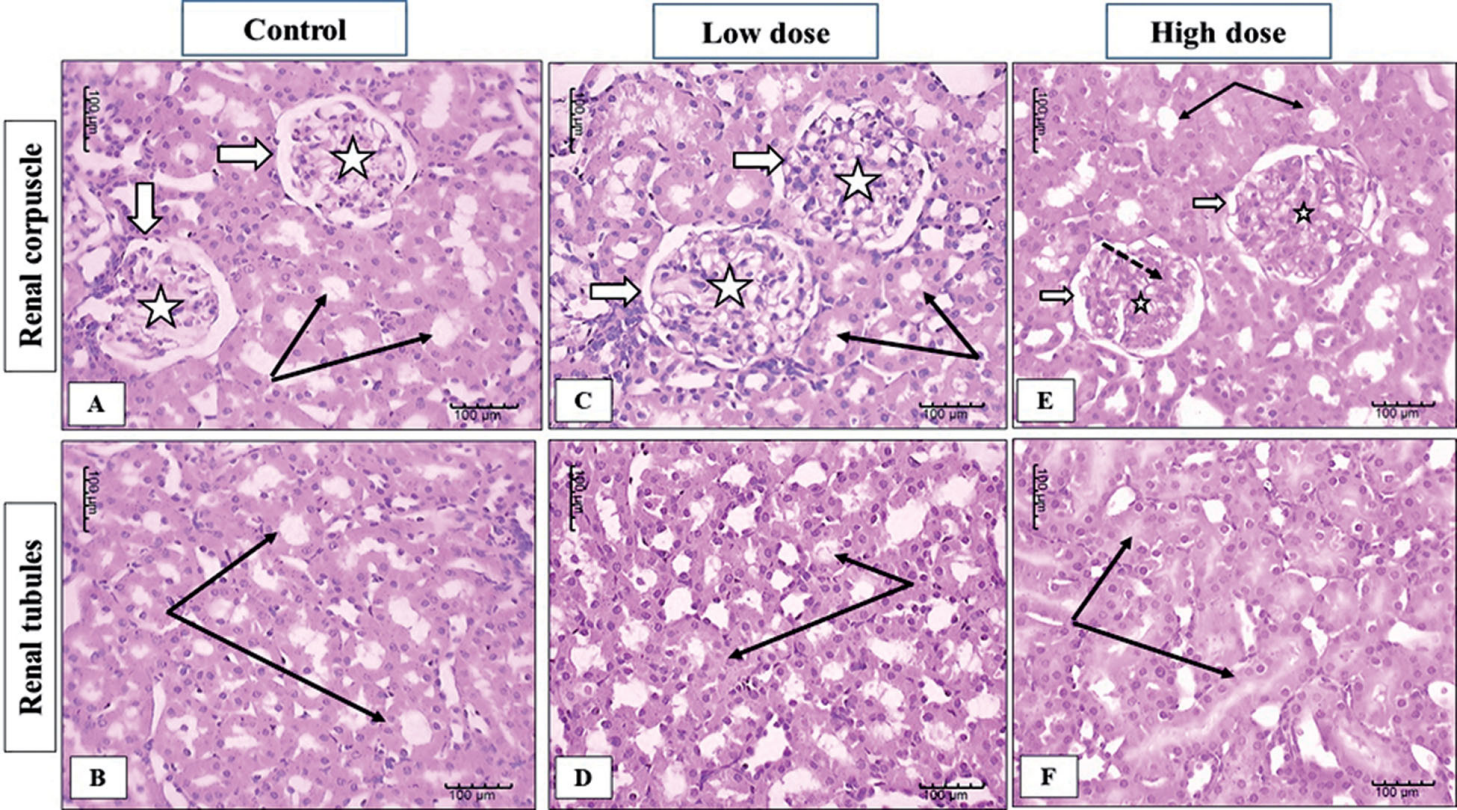
Illustrative photographs displaying the effect of low and high dose (100 and 200 mg/kg, respectively). MgO-zein nanowires on kidney histopathology in female rats (H & E stain × 200). Control group (A,B) showed normal renal corpuscles (white arrows) with its glomerular capillaries (stars), renal tubules showed intact normal cell lining (black arrows). Low dose group (C,D) showed an enlarged renal corpuscle with increase glomerular cellularity, no apparent change in renal tubules (black arrows). High dose group (E,F) showed normal renal corpuscle (white arrows) with a decrease in cellular density (stars) and increase mesangial substances (dotted arrows), renal tubules showed active large nuclei (black arrows).

The examination of the kidney sections from female rats showed normal kidney parenchyma in both cortical and medullary regions in the control group (Figure 7(A,B)). In the low dose (100 mg/kg) MgO-zein nanowires group slight enlargement of renal corpuscle with increase glomerular cellularity was observed while no apparent change in renal tubules was estimated (Figure 7(C,D)). High dose (200 mg/kg) MgO-zein nanowires group kidney showed normal renal corpuscles with a slight apparent decrease in cellular density and increased mesangial substances. Renal tubules showed active large nuclei (Figure 7(E,F)).

## Discussion

This study was designed to elucidate how far the different doses of MgO-zein nanowires are safe to be used as an anti-bacterial agent in the dental field. For this purpose, estimation of serum electrolytes, liver and kidney function parameters as well as the microscopic examination of the liver and kidney were achieved to reveal any abnormal changes.

In the current study, both liver and kidney were used to evaluate the cytotoxic effects of MgO nanowires as they represent the common organs affected by chemical toxicity. The liver is an important site for the breakdown of most metabolites in the body and is referred to as the “metabolic clearing house” of the body [32–34]. Also, kidneys are responsible for the filtration of the blood, so it is not surprising that deleterious agents in the blood may accumulate there [35].

Unfortunately, the wide use of different forms of metal oxide NPs in modern technology including medical systems, and their presence in the environment may lead to an increased risk of human exposure [36]. These NPs can intoxicate the body after invading it by various tracks due to their high reactivity and penetration capability into the living cells [37]. Indeed, the research found that the brain, liver, and lungs are the most vulnerable studied affected organs by NPs [38,39].

Accordingly, researchers have shown great interest in developing antibacterial active nanomaterials [40]. Lately, new and enhanced structural and physicochemical properties were generated by the production of one-dimensional nanomaterials, e.g. nanowires [41–43]. In this regard, the affirmation of nanoparticles biocompatibility and bio-safety by several toxicological investigations broaden the application of nanowires. These studies examined silver nanowires [44,45], zinc oxide nanowires [46], titanium dioxide nanowires [47], nickel nanowires [48], iron nanowires [49], tellurium nanowires [50], and cerium nanowire [51].

Recently, zein-based MgO nanowires were prepared by Naguib et al. [22] who examined the influence of the anionic charge effect of zein over the formation, physiochemical, *in-vitro* dissolution, and stability of MgO nanowires. The addition of zein was found to be very advantageous, where it was reported previously that the adsorbed zein on nanowires aided increased wettability of the high surface area of MgO, further increasing the ability of MgO to be dispersed in solvents, which will lead to the formation of the more homogenous nanowire. The release was also found prolonged about 12 h majorly due to the swelling mechanism of zein that could be advantageous in designing various dental formulations as reported [52]. Moreover, it was found that an electrostatic attraction occurred between positive and negative charges of MgO and zein, respectively, allowing efficient coating around the nanowires [29].

In the present study, we assessed the cytotoxic effects of low dose (100 mg/kg body weight/day) *vs.* high dose (200 mg/kg body weight/day) of Zein coated MgO nanowires. These doses were chosen based on our previous study [25]. On the same line, Mazaheri et al. examined at various concentrations (62.5–125–250–500 μg·mL^−1^) Mg O Nanospheres (non-coated) over a 28-days period in Wistar rat under *in vivo* conditions using hematological, biochemical, and histopathological studies. They showed that Mg oxide Nanospheres (non-coated) in concentrations lower than 250 μg·mL^−1^ were safe for desired applications [53].

It was stated that the dose conversion from animal to human studies is one of the most controversial areas in clinical pharmacology were understanding the concept of extrapolation of dose between species is important for pharmaceutical researchers [54,55]. Also, it was reported that the extrapolating nanomaterials disposition and pharmacokinetics from preclinical animal models to humans will hopefully result in a more straightforward roadmap for the clinical translation of promising nanomaterials [56]. In this concern, several equations were used to calculate the human equivalent dose (HED); most commonly was that related to body weight [55] as follows:

$$\mathrm{HED}\;(\mathrm{mg}/\mathrm{kg})\;=\text{ Animal dose }(\mathrm{mg}/\mathrm{kg})\;\times \;(\mathrm{Animal}\;K_{m}/\mathrm{Human}\;K_{m})$$

The correction factor (*K_m_*) is estimated by dividing the average body weight (kg) of species by its body surface area (m^2^).

In our study, the low animal dose is 100 mg/Kg while the high dose is 200 mg/Kg. As the average Rat *K_m_* is 6 and the average human *K_m_* is 37. The HED for the low dose would be 16.2 mg/Kg while the HED for the high dose would be 32.4 mg/Kg.

This study results showed that MgO-zein nanowires in the used doses did not affect the electrolytes levels compared to the control levels. In addition, both male and female rats have exhibited the same responses. Similar results previously reported that administration of various MgO preparations did not change serum potassium ion levels in healthy human volunteers [57]. It was reported that the electrolytes (electrolyte panel) contain many salts and minerals, like sodium, potassium, chloride, and bicarbonate, that are present in the blood and tissues [58]. They function to transfer electrical impulses across the body. Furthermore, electrolytes assist in transferring nutrients and cleaning the cells, preserve a normal water balance, and maintain normal pH [59]. Also, it was documented that any disease conditions that impact the quantity of fluid in the body like dehydration or influence the lung, kidney, metabolism, or breathing influence to induce fluid, electrolytes, or pH imbalance (acidosis or alkalosis). Electrolyte panels can be determined as important components of the basic metabolic panel. In addition, the determination of serum electrolyte concentration can aid to investigate if there is an electrolyte imbalance or not [14].

In the present study, both kidney and liver were chosen to appraise the toxicity of MgO-zein nanowires on their structure and function. The liver is one of the most prominent organs in which NPs accumulate, which may cause a change in its vital function. The high level of liver enzymes in the serum is one of the most important signs of liver cells injury damage, inflammation, or cholestasis [60]. Also, the kidney is a central organ that plays an important role in drugs elimination and toxicity. This makes it the most susceptible to drug toxicity. Thus, kidney safety is routinely evaluated during safety assessments of the drug during its preclinical stages [61]. Basically, blood urea nitrogen (BUN) and serum creatinine are the most applied tests upon which one can rely to monitor kidney function [62].

This investigation revealed that MgO-zein nanowires in both 100 and 200 mg/kg doses did not produce any effective alteration in liver function markers serum levels (ALT, AST, ALP, and bilirubin) in treated rats of both genders. The protected levels of these markers in this study resulted from the preserved integrity of the hepatocytes membranes which prevent the enzymes leakage to the bloodstream. Previous studies noticed that MgO NPs in both 250 and 500 mg/kg doses did not alter liver function markers in both genders (ALT and AST).

Also, in this study, it was noticed that MgO-zein nanowires in both 100 and 200 mg/kg doses did not produce any effective alteration in serum creatinine in treated rats of both genders. Interestingly, MgO-zein nanowires at 100 mg/kg significantly decreased BUN in both rats’ gender while MgO-zein nanowires at 200 mg/kg significantly decreased BUN in female rats only. These findings are in agreement with the study of Gelli et al. [13] who exhibited no possible toxicity in rats treated with low concentrations of MgO nanoparticles (62.5 and 125 μg·mL^−1^). A recent study also noticed that MgO NPs in dose below 250 µg/ml did not alter kidney function markers (urea and creatinine) in rats [53].

The microscopic results in this study revealed that low dose MgO-zein nanowires did not alter the hepatic and renal tissue histology which goes on hand with biochemical analysis for liver and kidney functions. However, mild alterations were observed in high dose MgO-zein nanowires as mild congestion in some sinusoids of the liver and proliferation of bile ductules; also, some kidney tubules looked dilated which may be due to increased blood flow and filtration rate. However, there were no major histological changes in both organs. It was demonstrated that congestion in the liver biopsy and dilatation of sinusoids result from impairment of venous outflow [63,64].

In conclusion, based on the observed results, zein-coated MgO NPs have no biochemical- or cytotoxicity of different doses both in male and female rats. We can recommend it to be utilized as an effective antimicrobial compound that can be combined in the preparation of a diversity of new dental formulations for the amelioration of dental care and oral health due to its safety and absent cytotoxic profile.

## Data Availability

Data are available from the corresponding author upon reasonable request.
